# Dietary Intake of Fatty Acids, Total Cholesterol, and Stomach Cancer in a Chinese Population

**DOI:** 10.3390/nu11081730

**Published:** 2019-07-26

**Authors:** Yu-Hui Zhu, Somee Jeong, Ming Wu, Zi-Yi Jin, Jin-Yi Zhou, Ren-Qiang Han, Jie Yang, Xiao-Feng Zhang, Xu-Shan Wang, Ai-Ming Liu, Xiao-Ping Gu, Ming Su, Xu Hu, Zheng Sun, Gang Li, Li-Ming Li, Li-Na Mu, Qing-Yi Lu, Jin-Kou Zhao, Zuo-Feng Zhang

**Affiliations:** 1 Department of Epidemiology, Fielding School of Public Health, University of California at Los Angeles (UCLA), Los Angeles, CA 90095, USA; 2 Department of Non-communicable Chronic Disease Control, Jiangsu Provincial Center for Disease Control and Prevention, Nanjing 210009, China; 3 Department of Epidemiology, School of Public Health, Fudan University, Shanghai 200032, China; 4 Ganyu County Center for Disease Control and Prevention, Ganyu 222100, China; 5 Dafeng County Center for Disease Control and Prevention, Dafeng 224100, China; 6 Chuzhou County Center for Disease Control and Prevention, Chuzhou 223200, China; 7 Tongshan County Center for Disease Control and Prevention, Tongshan 221006, China; 8 Department of Epidemiology and Biostatistics, Peking University School of Public Health, Beijing 100191, China; 9 Department of Social and Preventive Medicine, State University of New York at Buffalo, Buffalo, NY 14260, USA; 10 Center for Human Nutrition, Department of Medicine, UCLA David Geffen School of Medicine, University of California at Los Angeles (UCLA), Los Angeles, CA 90095, USA

**Keywords:** Case-control study, dietary fatty acids, dietary cholesterol, stomach cancer, China

## Abstract

To investigate the associations between dietary fatty acids and cholesterol consumption and stomach cancer (SC), we analyzed data from a population-based case-control study with a total of 1900 SC cases and 6532 controls. Dietary data and other risk or protective factors were collected by face-to-face interviews in Jiangsu Province, China, from 2003 to 2010. Adjusted odds ratios (ORs) and 95% confidence intervals (CIs) were estimated using multiple unconditional logistic regression models and an energy-adjusted method. The joint associations between dietary factors and known risk factors on SC were examined. We observed positive associations between dietary saturated fatty acids (SFAs), monounsaturated fatty acids (MUFAs), and total cholesterol and the development of SC, comparing the highest versus lowest quarters. Increased intakes of dietary SFAs (*p*-trend = 0.005; aOR, 1.11; 95% CI, 1.01–1.22 with a 7 g/day increase as a continuous variable) and total cholesterol (*p*-trend < 0.001; aOR, 1.13; 95% CI, 1.06–1.22 with a 250 mg/day increase as a continuous variable) were monotonically associated with elevated odds of developing SC. Our results indicate that dietary SFAs, MUFAs, and total cholesterol are associated with stomach cancer, which might provide a potential dietary intervention for stomach cancer prevention.

## 1. Introduction

Stomach cancer (SC) is ranked as the fifth-most commonly diagnosed cancer and the third leading cause of cancer deaths worldwide by GLOBOCAN 2018 [1]. In general, the incidence rate of SC is two to three times more common in men than in women. The incidence rates of SC in Eastern Asia are higher than the rates in Northern America, Northern Europe, and Africa. About 44% of the world’s total stomach cancer diagnosed, and almost half of the world’s total stomach cancer deaths (49.9%) occurred in China [1]. As the second-most common and the second-most deadly cancer in China [2], the age-adjusted 5-year survival of stomach cancer is relatively poor, in the range of 30.2–35.9% [3]. 

The established risk or protective factors for SC include *Helicobacter* (*H.*) *pylori* infection [4], tobacco smoking [5,6], heavy alcohol consumption [7], and some dietary factors [8]. The stomach is a part of the digestive system, which processes and digests foods by secreting acid and enzymes. Nutrients are absorbed in the small intestine, including vitamins, minerals, carbohydrates, fats, and proteins. Prior studies found that high intakes of dietary salt [9] and red meat [10] were associated with the development of stomach cancer, while non-starchy vegetables, fruits, and specific micronutrients (e.g., selenium, zinc), as well as green tea drinking, may be protective factors for the disease [11,12,13,14,15]. 

Until now, more than 20 types of fatty acids are found in food. Epidemiologic studies have examined the relationships between various dietary fatty acids consumption and SC. In a meta-analysis on the relationship between dietary intake of fat and SC with one cohort and 21 case-control studies, dietary intake of total fat was potentially associated with SC (highest vs. lowest OR = 1.18, 95% CI: 0.999–1.39) [16]. When separated into subtypes of fatty acids, some case-control studies found the development of SC has been positively linked to the dietary intakes of saturated fatty acids (SFAs) [17,18,19,20,21] and monounsaturated fatty acids (MUFAs) [17,19]. However, other case-control studies found no significant associations between dietary SFAs [22,23,24,25,26], MUFAs [18,23,25,26], PUFAs [18,19,20,23,25], and the development of stomach cancer. A cohort study among elderly individuals in the U.S. [27] found that dietary intakes of total fat and selected fat subtypes (SFAs, MUFAs, PUFAs, trans-fat, and n-3 PUFAs) were not related to the risk of SC. Furthermore, some previous case-control studies reported negative associations with dietary MUFAs [22] and PUFAs [17,22,28,29]. Similarly, there are inconsistent results on dietary cholesterol with the development of SC. Positive associations between intake of dietary cholesterol and the development of SC were reported in some studies [18,20,30], but not in others [28,31]. 

Since most of the previous epidemiological studies had relatively small sample sizes and did not properly adjust for *H. pylori* infection or other established risk factors, the reported associations might be either underpowered or confounded. We tested the hypotheses and evaluated associations of total and subtypes of fatty acids, as well as total cholesterol on the development of SC using the data from a large population-based case-control study in a Chinese population. 

## 2. Materials and Methods

### 2.1. Study Population

A population-based case-control study, named Jiangsu Four Cancers (JFC) study, was conducted in four counties (Dafeng, Ganyu, Chuzhou, Tongshan) in Jiangsu Province, China, to study risk and protective factors of four top cancers (lung, stomach, esophageal, and liver cancers). The methodological details and rationales of the JFC study have been published elsewhere [32]. In short, primary stomach cancer cases were identified from cancer registries of the County Center for Disease Control and Prevention (CDC) from January 2003 to December 2010. The control group was randomly chosen from the list of residence registry of each county and initially matched to the corresponding cases for age (±five years) and gender. All participants were restricted to residents who had lived in the county for more than five years before the date of stomach cancer diagnosis for the case group or before the interview date for the control group. We pooled all controls for four types of cancers together in order to increase the sample size of the control group. A total of 2216 stomach cancer cases and 8019 controls were recruited (Supplemental Table S1). The JFC study was approved by both Jiangsu CDC and UCLA institutional review boards. Written informed consents were obtained from all participants before the epidemiologic data and biological specimen collections. 

### 2.2. Data Collection

Face-to-face interviews of participants were conducted using a structured questionnaire with detailed information on (1) demographic features (age, gender, education, family income per year, and home address, etc.); (2) residence environment factors; (3) health behaviors (dietary history, life-long history of tobacco smoking, alcohol consumption, green tea drinking, and physical activity); (4) medical history; (5) occupational exposures; (6) family history of cancer; and (7) reproductive factors among women. The interviews of the cases took place at their hospital ward or home after they were reported to the county cancer registry, and those of the controls took place at their home.

Participants were asked to report their general dietary history one year before the diagnosis or the interview date to capture dietary patterns by using a 90-item food frequency questionnaire (FFQ). For each food item, participants were asked whether they ever consumed the food or not in the year before the diagnosis or the interview date. For each food item they consumed, the frequency and portion size were asked, according to four predefined frequency categories (times per year, times per month, times per week, and times per day) and one predefined portion size (1 Liang = 50 g). An average daily intake of each food item was calculated by multiplying the portion size and frequency of consumption per day. 

Non-fasting peripheral blood samples (5–8 mL) were collected after the time of the interview. Anti-*H. pylori* antibody immunoglobulin G (anti-*H. pylori* Ab IgG) was measured among the cases and the controls using enzyme-linked immunosorbent assays (ELISA) with kits from Beier Bioengineering (Beijing, China). According to the manufacturer’s instructions, levels of IgG were categorized as seropositive and seronegative for *H. pylori* infection according to the cutoff value. 

### 2.3. Dietary Assessments

For each food item in FFQ, a matched food item or list of food items were found in the China Food Composition (CFC) Tables 2010, released by the China CDC (Institute of Nutrition and Food Safety 2010). For two food items in FFQ could not be matched with the CFC tables, frog and sugar cane, we employed the Japanese Food Composition Tables for frog and the U.S. Department of Agriculture (USDA) database for sugar cane. The average daily intake of calories for each food item was estimated by multiplying the average daily intake of each food by the corresponding calorie value obtained from the CFC Tables. Then, the total intake of calories per day was calculated by summing up the calories from all the food items the participants consumed. In the JFC study population, the median of total intake of calories from food among controls was 1855.0 calories per day. We excluded individuals who consumed less than 500 or more than 5000 calories per day, and those who ate only less than four food items (cases = 316, controls = 1487) because their FFQs were considered incomplete. Finally, 1900 SC cases and 6532 controls remained in our analyses (see Supplemental Figure S1). 

We multiplied the average daily intake of each food by the corresponding contents of fatty acids obtained from the CFC Tables and summed up the values for each participant. Intakes of total fatty acids (FAs), saturated fatty acids (SFAs), monounsaturated fatty acids (MUFAs), and polyunsaturated fatty acids (PUFAs) were estimated. For PUFAs, n-3 fatty acids, including alpha-linolenic acid (ALA), docosahexaenoic acid (DHA), eicosapentaenoic acid (EPA); and n-6 fatty acids, including linoleic acid (LA) and arachidonic acid (AA) were estimated. We included total fat and cholesterol in data analyses. We further evaluated the impact of the cumulative ingestion of each type of fatty acids and total cholesterol on the susceptibility of stomach cancer.

### 2.4. Statistical Analysis

A chi-square test or *t*-test was used to compare the distribution of potential risk and protective factors between cases and controls. The associations between dietary fatty acids, total cholesterol, and SC, were evaluated and adjusted odds ratios (aORs) and their 95% confidence intervals (CIs) were estimated using multiple unconditional logistic regression models. Potential confounding factors included age (years), gender (male vs. female), county (Dafeng, Ganyu, Chuzhou, Tongshan), education (illiterate, primary, middle, high school or above), income 10 years ago (<1000, 1000 to <1500, 1500 to <2500, ≥2500 yuan/year), family history of stomach cancer (yes vs. no), tobacco smoking (yes or no and pack-years), alcohol consumption (ethanol, g/day), total energy intake (kcal/day), dietary sodium intake (<0.55, 0.55 to <1.04, 1.04 to <1.96, ≥1.96 g/day), *H. pylori* infection (yes vs. no), physical activity 10 years ago (yes vs. no), and body mass index (BMI) (<18.5, 18.5 to <24, 24 to <28, ≥28 kg/m^2^). To reduce extraneous variations, we analyzed dietary intakes of fatty acids and cholesterol adjusted for total energy intake using the residual method [33]. Adjusted ORs and 95% CIs of energy-adjusted dietary factors for SC were calculated by multiple unconditional logistic regression models. Dietary intakes of fatty acids and cholesterol were examined as both categorical variables and continuous variables. Dietary intakes of these nutrients were categorized as quartiles, according to their distributions among controls. Trend analyses were performed by scoring the ordinal level of dietary exposures (0, 1, 2, 3) and treating them as a continuous variable in the models. For continuous variables, the rescaling units were chosen based on the interquartile range (IQR) of controls as well as on the availability of intervention ranges. The 2016 Chinese Dietary Guidelines [34] recommended no more than 25 g ethanol/day for men and 15 g ethanol/day for women. Three groups of alcohol consumption were created by the recommendation: never (0 g ethanol/day), low-risk (≤25 g ethanol/day for men and ≤15 g ethanol/day for women), and high-risk (>25 g ethanol/day for men and >15 g ethanol/day for women).

The interactions among selected dietary factors (SFAs, MUFAs, and total cholesterol) and known risk factors, including tobacco smoking, alcohol drinking, *H. pylori* infection, dietary sodium intake, and family history of stomach cancer, were assessed based on the additive and multiplicative scales [35]. Stratified analyses of these factors were also conducted. The medians in controls were applied to dichotomize dietary ingestion of SFAs (7.14 g/day), MUFAs (9.85 g/day), and total cholesterol (207.21 mg/day) in both interaction and stratified analyses. Covariates considered as potential confounders or effect measure modifiers for analyses were identified based on a priori knowledge of the risk and protective factors for stomach cancer and by using a directed acyclic graph (DAG). 

Furthermore, sensitivity analyses were conducted regarding potential selection bias, multiple comparison issues, and imputations of missing data. First, we analyzed data based on a direct interview by excluding the data obtained by proxy interviews (Supplemental Table S2). We also excluded participants with reported total energy intake in the upper and lower 2.5% of values to this study [36] (cases = 309, controls = 1421) in data analyses to test for potential selection bias due to different exclusion criteria of total energy intake (Supplemental Table S3). To reduce potential false positive or inflated coefficient estimates of multiple comparisons, we used a semi-Bayes shrinkage method [37], leading to more conservative measurements (Supplemental Table S4). A total of 19% of participants did not have values for the *H. pylori* infection test, and total missing values for all variables were more than 20%. Multiple imputations of the Markov chain Monte Carlo (MCMC) method were used to impute values of each covariate in the full dataset (cases = 2216, controls = 8019) to maximize the use of available information if missing data are at random. We applied PROC MI (analysis of imputed data sets) in the SAS program (version 9.4, Cary, NC, USA) to specify the imputation model, and created ten imputed datasets. The variables in the imputation model included the outcome, and the covariates were the same as in the logistic regression models (Supplemental Table S4). Similar patterns of estimated ORs and 95% CIs were found in the multiple logistic regression models using the standard method, energy-adjusted method, semi-Bayes shrinkage method, and multiple imputation method. Therefore, we only present the results using the standard approach and the energy-adjusted method. All analyses were conducted using SAS. 

## 3. Results

The distributions of demographic characteristics, behavioral variables, total energy intake, and *H. pylori* infection among the stomach cancer cases (*n* = 1900) and controls (*n* = 6532) are summarized in Table 1. In brief, the cases and controls had similar distributions of gender, age, physical activity 10 years ago, and total energy intake. However, clear differences were observed regarding the county of residence, education level, income 10 years ago, body mass index (BMI), dietary sodium intake, tobacco smoking, alcohol consumption, family history of SC, and *H. pylori* infection. Compared to the control group, the cases were more likely to have a lower education level, lower income 10 years ago, and lower BMI level. The cases had the higher intake of dietary sodium and pack-year of tobacco smoking. Also, the proportions of high-risk drinking status, *H. pylori* infection, and having a family history of SC among the cases were higher than those of the controls. 

Table 2 shows the median values of dietary fatty acids and total cholesterol intake among the controls in this population using both non-adjusted and energy-adjusted methods. The median intakes of total fatty acids were 24.75 g/day for non-adjusted and 36.66 g/day for energy-adjusted methods. SFAs (median, 7.14 g/day for non-adjusted vs. 10.19 g/day for energy-adjusted), MUFAs (median, 9.85 g/day for non-adjusted vs. 15.41 g/day for energy-adjusted), and PUFAs (median, 6.93 g/day for non-adjusted vs. 9.64 g/day for energy-adjusted) were the main contributors to total fatty acids. n-3 (median, 0.96 g/day for non-adjusted vs. 1.59 g/day for energy-adjusted) and n-6 PUFAs (median, 5.97 g/day for non-adjusted vs. 8.05 g/day for energy-adjusted) were main contributors to PUFAs in this population. LA (median, 5.50 g/day for non-adjusted vs. 7.17g/day for energy-adjusted) was mainly consumed as n-6 PUFAs, and ALA (median, 0.93 g/day for non-adjusted vs. 1.53 g/day for energy-adjusted) was mainly consumed as n-3 PUFAs. The median ratio of n-3 and n-6 PUFAs was 0.16 for the non-adjusted method and 0.20 for the energy-adjusted method. The median of dietary cholesterol was 207.21 mg/day for the non-adjusted method and 161.49 mg/day for the energy-adjusted method.

The associations between dietary fatty acids and total cholesterol intakes based on the quartile distribution and SC are presented in Table 3. A positive association between dietary intake of total cholesterol and SC was observed comparing the highest quartile to the lowest quartile in the standard logistic regression model (aOR, 1.57; 95% CI, 1.26–1.96) and in the energy-adjusted model (adjusted odds ratios with residual method (rOR), 1.56; 95% CI, 1.23–1.93). Increased dietary intake of total cholesterol showed consistent dose-response associations with the increased odds of developing SC in both the standard logistic regression model and energy-adjusted model. Nevertheless, there was a weak or non-linear relationship between total fatty acids and SC. Among the subtypes of dietary fatty acids, dietary SFAs were positively associated with SC (*p*-trend = 0.005; aOR, 1.11; 95% CI, 1.01–1.22 with 7 g/day increments as a continuous variable). Dietary MUFAs were positively associated with SC as a categorical variable, but a null association was observed as a continuous variable. We did not observe clear associations between dietary intake of PUFAs or their subtypes and stomach cancer.

The potential interactions were evaluated between dietary intake of SFAs, MUFAs, and total cholesterol (high vs. low) and known risk factors on stomach cancer, and results were shown in Table 4. After adjusting for potential confounding factors, we observed dietary intake of SFAs interacted with tobacco smoking (the ratio of odds ratio (ROR), 0.76; 95% CI, 0.59–0.99), alcohol drinking (ROR, 0.75; 95% CI, 0.57–0.98), and dietary sodium intake (ROR, 0.75; 95% CI, 0.57–0.99) on the development of stomach cancer at the multiplicative scale of the standard model. Multiplicative interaction between dietary MUFAs and dietary sodium intake was also observed (ROR, 0.70; 95% CI, 0.53–0.92). However, we did not identify obvious interactions in the energy-adjusted models. 

In Table 5, we detected heterogeneity of the associations between dietary SFAs and SC across tobacco smoking (*p* for heterogeneity = 0.04), alcohol drinking (*p* for heterogeneity = 0.03), and dietary sodium intake strata (*p* for heterogeneity = 0.04) in the standard models, which is consistent with our observed associations in Table 4. Similarly, heterogeneity of the association between dietary MUFAs and SC across dietary sodium intake strata was observed (*p* for heterogeneity = 0.01). Nonetheless, there was no clear heterogeneity between dietary SFAs, MUFAs, total cholesterol, and SC across the strata of these risk factors in the energy-adjusted models. 

## 4. Discussion

In this study, we have observed that higher intakes of dietary SFAs, MUFAs, and total cholesterol were associated with the development of stomach cancer. The associations were strong with a dose-response pattern. However, no obvious dose-response relationships were observed between the consumptions of total fatty acids, PUFAs and their subtypes, and SC. And no consistent interaction or heterogeneity of the associations was identified between SFAs, MUFAs, total cholesterol, and known risk factors on SC. 

Epidemiological and experimental studies have suggested that different subtypes of fatty acids appear to play some roles in the carcinogenesis and the development of stomach cancer [38]. Several case-control studies have reported that dietary SFAs were positively associated with stomach cancer [17,18,19,20,21], which is consistent with our results. However, other case-control studies [22,23,24,25,26] and one cohort study [27] reported null associations with SFAs. In this study, high intake of dietary MUFAs showed a positive association with SC, consistent with two previous studies [17,19]. Nevertheless, the intake of vegetable oils, which is rich in oleic acid, has been inversely associated with SC in three case-control studies [23,24,39]. The conflicting findings might be associated with a limited sample size and insufficient adjustment for potential confounding factors and might also be related to the complex composition of MUFAs. It has been suggested that the various sources of MUFAs, animal fat, and vegetable oils may differentially affect the association between MUFAs intake and SC [16]. Given that foods are consumed in combination, variations of MUFAs are highly correlated with other nutrients, making it difficult to distinguish their individual effects [40]. 

PUFAs, which are involved in many critical biological functions, are essential nutrients for life, which cannot be produced endogenously [41]. However, very few studies have comprehensively investigated the intakes of all PUFA subtypes, n-3 and n-6 PUFAs in particular. In this study, we have included most of PUFA subtypes and found, when the subtypes of PUFAs were separated, neither n-6 PUFAs (including LA and AA) nor n-3 PUFAs (including ALA, EPA, and DHA) were associated with SC. Like MUFAs, the different sources of PUFAs might be related to the inconsistent results of PUFAs on SC. Thiébaut et al. [42] reported that high consumption of alpha-linolenic acid (ALA) from fruit and vegetables was inversely associated with breast cancer, but ALA from nut mixes and processed meat was positively related to the disease. PUFAs may also be related to carcinogenic compounds accumulated along the food chain in the main source of dietary PUFAs [43]. In our study, we found that the consumption of fresh fish among the cases was higher than those among the controls, which might be confounded by other factors, such as rich toxins in fresh fish due to water contamination. Therefore, carefully identifying the sources of dietary fatty acids and minimizing the effects of confounding factors are necessary and essential for evaluating the association between subtypes of dietary fatty acids and SC. 

Our finding suggests that a high intake of dietary cholesterol may potentially increase the odds of stomach cancer, which is consistent with three previous case-control studies [18,20,30]. However, no clear association was discovered in a hospital-based case-control study in Italy (OR, 1.11; 95% CI, 0.94–1.32) [28] and a population-based case-control study in Poland (OR, 0.90; 95%CI, 0.58–1.38) [31]. Most of the prior studies on dietary cholesterol with SC have been conducted in western countries, where the incidence of SC is relatively low [1], therefore, the power of these studies was low due to relatively small number of stomach cancer cases, leading to inconsistent findings. The biological mechanisms on the relationship between dietary intake of cholesterol and stomach cancer have been hypothesized. Controlled experiments in mice suggest an association between dietary cholesterol and cancer [44,45,46,47]. Hypercholesterolemia, associated with high cholesterol intake, might be linked to elevated inflammatory activity, which may play a role in cancer development [48]. Jung et al. [49] also emphasized that hypercholesterolemia was a risk factor for the occurrence of gastric dysplasia. A preclinical study reported that cholesterol metabolism might play an important role in *H. pylori* eradication [50], however, we did not observe any effect measure modification between dietary cholesterol and *H. pylori* infection on stomach cancer.

When we assessed the effect modification of SFAs, MUFAs, and total cholesterol with tobacco smoking, alcohol drinking, *H. pylori* infection, dietary sodium intake, and family history of stomach cancer for the disease, no consistent interactions were identified. The only published paper on the effect modification of fatty acids by smoking, alcohol, and BMI in the U.S. population [27] concluded that there was no clear effect modification of dietary fatty acids intake by tobacco smoking and alcohol drinking on the development of the disease, which is consistent with our results. 

The total number of male participants is more than female participants in our study, which probably reflects the difference in incidence between males and female. From GLOBOCAN 2018, SC incidences are about 32.1 per 100,000 for men and 13.2 per 100,000 for women in Eastern Asia, which is consistent with our study [1]. The differences in lifestyle factors, such as diet and smoking, as well as probable hormonal factors, may explain gender heterogeneity. Future research may focus on the interactions between gender or hormonal factors and established risk factors on the development of the disease.

The strengths of this study include a population-based study design, extensive epidemiologic data including *H. pylori* infection status and dietary habits, and a large sample size which allow us to examine both main associations and interactions. However, some potential limitations of the study should be addressed. Although we collected dietary history one year before the diagnosis for cases and one year before the interview for controls, the cases might have already changed their dietary pattern a year before their diagnosis. There might be the possibility of reverse causality between dietary factors and stomach cancer. However, the majority of dietary fatty acids and cholesterol were from high-fat foods. These foods might potentially result in stomach upset or gastric reflex, especially in stomach cancer cases if they had early gastric symptoms, leading to reduced intake of foods with high fatty acids and cholesterol. If reverse causality does exist, we might observe the inversed association. Based on the observed positive associations with fatty acids and cholesterol, the possibility of reverse causality may be minimal. 

As with other case-control studies, measurement bias and selection bias are also potential limitations. The estimations of dietary intake of fatty acids and cholesterol, just like measurements of other nutrients, are prone to measurement bias. The conversion of food items into related nutrients is complex, which may lead to measurement errors. To reduce measurement errors, we mainly used the China Food Composition Tables 2010 to calculate total energy intake and nutrients. For food items that contained multiple foods (e.g., beef and mutton), we weighted certain foods to reflect more common consumption or to reflect preparation methods in the population. We also used residual energy adjustment in the logistic regression models. The results were consistent with those in the standard logistic regression models. 

In this study, the participation rates were 40% among the SC cases and 87% among the controls, which might lead to potential selection bias. The reason for the low rate is that SC cases diagnosed at advanced stages were too ill to enroll. Among the recruited cases and controls, the exclusion of participants who had missing data for diet and other covariates might result in selection bias if data are not missing completely at random. Hence, sensitivity analyses were performed to test whether excluding participants with an extreme energy intake would cause potential selection bias. Moreover, multiple imputations were used in the dietary analyses to compare the imputed estimates against the complete case analysis. However, we did not find the obvious inconsistent associations when results from the main analyses were compared with from sensitivity analyses. For confounding bias, we adjusted for potential confounding factors in all analyses based on prior knowledge and confounding assessment. 

## 5. Conclusions

The findings from this study suggest positive associations between high intakes of dietary SFAs, MUFAs, total cholesterol and the development of stomach cancer in a large population-based case-control study in China. The findings might shed some light on potential etiological roles of dietary fatty acids and cholesterol on stomach cancer, and consequently, the possible dietary intervention could be implemented to prevent stomach cancer in Chinese population.

## Figures and Tables

**Table 1:** Demographic characteristics of stomach cancer and population controls among participants (cases = 1900, controls = 6532).

Variables	Stomach Cancer (*n* = 1900) *n* (%)	Controls (*n *= 6532) *n* (%)	*p*-Value ^a^
Study area			
Dafeng	641 (33.7)	2508 (38.4)	
Ganyu	527 (27.7)	1872 (28.7)	
Chuzhou	454 (23.9)	1109 (17.0)	
Tongshan	278 (14.6)	1043 (16.0)	<0.001
Gender			
Male	1401 (73.7)	4713 (72.2)	
Female	499 (26.3)	1819 (27.9)	0.17
Age			
Mean (SD)	64.1 (10.8)	64.0 (11.3)	0.73
<50	185 (9.7)	699 (10.7)	
50 to <60	407 (21.4)	1450 (22.2)	
60 to <70	652 (34.3)	2109 (32.3)	
≥70	656 (34.5)	2274 (34.8)	0.31
Education level			
Illiterate	954 (50.2)	3215 (49.2)	
Primary school	663 (34.9)	2027 (31.0)	
Middle school	224 (11.8)	1007 (15.4)	
High school or above	55 (2.9)	270 (4.1)	
Missing	4 (0.2)	13 (0.2)	<0.001
Income 10 years ago (Yuan/year)			
<1000	465 (24.5)	1393 (21.3)	
1000 to <1500	383 (20.2)	1218 (18.7)	
1500 to <2500	496 (26.1)	1707 (26.1)	
≥2500	502 (26.4)	2116 (32.4)	
Missing	54 (2.8)	98 (1.5)	<0.001
Body mass index (kg/m^2^)			
<18.5	298 (15.7)	410 (6.3)	
18.5 to <24	1277 (67.2)	3970 (60.8)	
24 to <28	255 (13.4)	1743 (26.7)	
≥28	61 (3.2)	372 (5.7)	
Missing	9 (0.5)	37 (0.6)	<0.001
Exercise 10 years ago			
No	1304 (68.6)	4564 (69.9)	
Yes	596 (31.4)	1968 (30.1)	0.3
Total energy intake (kcal/day)			
<1549.3	433 (22.8)	1633 (25.0)	
1549.3 to <2036.8	487 (25.6)	1633 (25.0)	
2036.8 to <2624.6	468 (24.6)	1633 (25.0)	
≥2624.6	512 (27.0)	1633 (25.0)	0.14
Dietary sodium intake (g/day)			
<0.55	367 (19.3)	1633 (25.0)	
0.55 to <1.04	504 (26.5)	1633 (25.0)	
1.04 to <2.00	479 (25.2)	1633 (25.0)	
≥2.00	550 (29.0)	1633 (25.0)	<0.001
Tobacco smoking			
Never	734 (38.6)	3136 (48.0)	
Ever	1166 (61.4)	3396 (52.0)	<0.001
Pack-years of tobacco smoking			
0	734 (38.6)	3136 (48.0)	
1 to <20	219 (11.5)	746 (11.4)	
20 to <40	303 (16.0)	1028 (15.7)	
≥40	477 (25.1)	1139 (17.4)	
Missing	167 (8.8)	483 (7.4)	<0.001
Alcohol drinking			
Never	903 (47.5)	3223 (49.3)	
Ever	997 (52.5)	3309 (50.7)	0.02
Grams ethanol/day in the 1990s ^b^			
Never	1034 (54.4)	3692 (56.5)	
Low–risk	132 (7.0)	495 (7.6)	
High–risk	705 (37.1)	2209 (33.8)	
Missing	29 (1.5)	136 (2.1)	0.03
Family history of stomach cancer			
No	1711 (90.1)	6170 (94.5)	
Yes	189 (10.0)	362 (5.5)	<0.001
*H. pylori* infection			
Negative	332 (17.5)	1613 (24.7)	
Positive	1062 (55.9)	3811 (58.3)	
Missing	506 (26.6)	1108 (17.0)	<0.001

^a^ Based on the chi-square test; *t*-test for the mean. ^b^ Never (0 g ethanol/day); low-risk drinking: men (≤25 g ethanol/day) and women (≤15 g ethanol/day); high-risk drinking: men (>25 g ethanol/day) and women (>15 g ethanol/day) based on the 2016 Chinese Dietary Guidelines.

**Table 2:** The medians of dietary fatty acids and cholesterol intakes in controls with a normal range of total energy intake (*n* = 6532).

	Non-Energy Adjusted		Energy-Adjusted (Residual Method)	
Variables	Median	Quartile 1–Quartile 3	Median	Quartile 1–Quartile 3
Total fatty acids (g/day)	24.75	14.85–40.17	36.66	27.20–47.48
SFAs (g/day)	7.14	4.35–11.56	10.19	7.59–13.33
MUFAs (g/day)	9.85	5.57–16.65	15.41	11.16–20.56
PUFAs (g/day)	6.93	4.28–10.80	9.64	7.38–12.21
n-6 PUFAs (g/day)	5.97	3.76–9.14	8.05	6.20–10.08
LA (g/day)	5.50	3.49–8.35	7.17	5.58–9.03
AA (g/day)	0.38	0.18–0.78	0.73	0.51–1.03
n-3 PUFAs (g/day)	0.96	0.54–1.70	1.59	1.16–2.12
ALA (g/day)	0.93	0.52–1.64	1.53	1.13–2.05
EPA (mg/day)	10.47	4.30–21.77	19.06	12.47–27.42
DHA (mg/day)	9.97	2.52–24.49	25.96	17.52–37.34
n-3/n-6 PUFAs ratio	0.16	0.13–0.20	0.20	0.18–0.22
Total fat (g/day)	31.07	18.84–48.98	43.39	31.67–57.20
Total cholesterol (mg/day)	207.21	107.24–352.09	161.49	65.98–298.38

Notes: SFAs: saturated fatty acids; MUFAs: monounsaturated fatty acids; PUFAs: polyunsaturated fatty acids; n-6 PUFAs: including LA and AA; LA: linoleic acid; AA: arachidonic acid; n-3 PUFAs: including ALA, EPA, and DHA; ALA: α-linolenic acid; EPA: eicosapentaenoic acid; DHA: docosahexaenoic acid.

**Table 3:** The associations between dietary fatty acids and total cholesterol intakes and stomach cancer among individuals with a normal range of total energy intake (cases = 1900, controls = 6532).

Variables	Cases/Controls	aOR (95% CI) ^a^	rOR (95% CI) ^a^
Total fatty acid (g/day)			
Q1 (<14.85)	356/1633	1.00	1.00
Q2 (14.85 to <24.75)	431/1633	1.28 (1.04, 1.59)	1.20 (0.98, 1.47)
Q3 (24.75 to <40.17)	581/1633	1.58 (1.27, 1.97)	1.39 (1.13, 1.70)
Q4 (>40.17)	532/1633	1.44 (1.10, 1.87)	1.18 (0.95, 1.45)
*p*-value for trend		0.002	0.08
Per 25 g increase ^b^		1.09 (0.99, 1.19)	
SFAs (g/day)			
Q1 (<4.35)	361/1633	1.00	1.00
Q2 (4.35 to <7.14)	438/1633	1.26 (1.02, 1.56)	1.20 (0.98, 1.46)
Q3 (7.14 to <11.56)	566/1633	1.48 (1.19, 1.85)	1.37 (1.12, 1.69)
Q4 (>11.56)	535/1633	1.42 (1.09, 1.84)	1.29 (1.04, 1.59)
*p*-value for trend		0.005	0.01
Per 7 g increase		1.11 (1.01, 1.22)	
MUFAs (g/day)			
Q1 (<5.57)	356/1633	1.00	1.00
Q2 (5.57 to <9.85)	435/1633	1.19 (0.96, 1.47)	1.14 (0.93, 1.39)
Q3 (9.85 to <16.65)	572/1633	1.50 (1.20, 1.86)	1.43 (1.16, 1.76)
Q4 (>16.65)	537/1633	1.41 (1.09, 1.82)	1.17 (0.95, 1.45)
*p*-value for trend		0.002	0.05
Per 10 g increase		1.05 (0.97, 1.14)	
PUFAs (g/day)			
Q1 (<4.28)	365/1633	1.00	1.00
Q2 (4.28 to <6.93)	461/1633	1.21 (0.98, 1.50)	1.17 (0.96, 1.43)
Q3 (6.93 to <10.80)	560/1633	1.47 (1.17, 1.85)	1.20 (0.97, 1.47)
Q4 (>10.80)	514/1633	1.16 (0.88, 1.52)	1.15 (0.93, 1.41)
*p*-value for trend		0.16	0.23
Per 6 g increase		1.09 (0.99, 1.20)	
n-6 PUFAs (g/day)			
Q1 (<3.76)	372/1633	1.00	1.00
Q2 (3.76 to < 5.97)	470/1633	1.23 (1.00, 1.52)	1.14 (0.94, 1.39)
Q3 (5.97 to < 9.14)	538/1633	1.36 (1.08, 1.71)	1.08 (0.88, 1.32)
Q4 (>9.14)	520/1633	1.18 (0.90, 1.55)	1.15 (0.94, 1.41)
*p*-value for trend		0.21	0.28
Per 5 g increase		1.10 (1.00, 1.21)	
LA (g/day)			
Q1 (<3.49)	380/1633	1.00	1.00
Q2 (3.49 to <5.50)	474/1633	1.25 (1.01, 1.54)	1.14 (0.94, 1.39)
Q3 (5.50 to <8.35)	522/1633	1.26 (1.00, 1.58)	1.06 (0.86, 1.31)
Q4 (>8.35)	524/1633	1.18 (0.90, 1.55)	1.14 (0.93, 1.40)
*p*-value for trend		0.31	0.32
Per 5 g increase		1.11 (1.00, 1.23)	
AA (g/day)			
Q1 (<0.18)	394/1633	1.00	1.00
Q2 (0.18 to <0.38)	438/1633	1.12 (0.91, 1.38)	1.08 (0.88, 1.32)
Q3 (0.38 to <0.78)	553/1633	1.26 (1.03, 1.56)	1.26 (1.03, 1.56)
Q4 (>0.78)	515/1633	1.20 (0.95, 1.52)	1.05 (0.86, 1.30)
*p*-value for trend		0.08	0.40
Per 1 g increase		1.01 (0.90, 1.14)	
n-3 PUFAs (g/day)			
Q1 (<0.54)	378/1633	1.00	1.00
Q2 (0.54 to <0.96)	417/1633	1.11 (0.90, 1.37)	1.17 (0.95, 1.43)
Q3 (0.96 to <1.70)	595/1633	1.48 (1.19, 1.84)	1.31 (1.07, 1.61)
Q4 (>1.70)	510/1633	1.18 (0.92, 1.53)	1.10 (0.89, 1.35)
*p*-value for trend		0.05	0.29
Per 1 g increase		1.04 (0.96, 1.13)	
ALA (g/day)			
Q1 (<0.52)	380/1633	1.00	1.00
Q2 (0.52 to <0.93)	420/1633	1.10 (0.89, 1.35)	1.14 (0.93, 1.40)
Q3 (0.93 to <1.64)	589/1633	1.46 (1.18, 1.82)	1.30 (1.06, 1.60)
Q4 (>1.64)	511/1633	1.17 (0.91, 1.50)	1.07 (0.87, 1.32)
*p*-value for trend		0.06	0.36
Per 1 g increase		1.04 (0.96, 1.13)	
EPA (mg/day)			
Q1 (<4.30)	398/1633	1.00	1.00
Q2 (4.30 to <10.47)	503/1633	1.20 (0.98, 1.48)	0.94 (0.77, 1.14)
Q3 (10.47 to <21.77)	450/1633	1.10 (0.88, 1.37)	0.99 (0.80, 1.22)
Q4 (>21.77)	549/1633	1.18 (0.93, 1.49)	1.09 (0.89, 1.33)
*p*-value for trend		0.37	0.32
Per 20 mg increase		1.00 (0.96, 1.03)	
DHA (mg/day)			
Q1 (<2.52)	423/1633	1.00	1.00
Q2 (2.52 to <9.97)	448/1633	1.13 (0.91, 1.39)	0.85 (0.70, 1.05)
Q3 (9.97 to <24.49)	443/1633	1.02 (0.81, 1.29)	0.83 (0.67, 1.03)
Q4 (>24.49)	586/1633	1.35 (1.06, 1.72)	1.14 (0.93, 1.40)
*p*-value for trend		0.03	0.14
Per 20 mg increase		1.04 (1.01, 1.08)	
n-3/n-6 PUFAs			
Q1 (<0.13)	406/1633	1.00	1.00
Q2 (0.13 to <0.16)	449/1633	1.00 (0.82, 1.23)	0.88 (0.72, 1.08)
Q3 (0.16 to <0.20)	535/1633	1.28 (1.05, 1.56)	1.11 (0.90, 1.34)
Q4 (>0.20)	510/1633	1.13 (0.92, 1.39)	1.09 (0.90, 1.32)
*p*-value for trend		0.07	0.14
Per 0.1 increase		1.12 (0.98, 1.27)	
Total fat (g/day)			
Q1 (<18.84)	355/1633	1.00	1.00
Q2 (18.84 to <31.07)	463/1633	1.31 (1.06, 1.61)	1.17 (0.96, 1.43)
Q3 (31.07 to <48.98)	557/1633	1.43 (1.15, 1.78)	1.31 (1.06, 1.61)
Q4 (>48.98)	525/1633	1.30 (1.00, 1.69)	1.10 (0.89, 1.38)
*p*-value for trend		0.04	0.28
Per 30 g increase		1.04 (0.95, 1.15)	
Total cholesterol (mg/day)			
Q1 (<107.24)	407/1633	1.00	1.00
Q2 (107.24 to <207.21)	429/1633	1.06 (0.87, 1.29)	1.17 (0.96, 1.42)
Q3 (207.21 to <352.09)	498/1633	1.32 (1.08, 1.61)	1.45 (1.18, 1.77)
Q4 (>352.09)	566/1633	1.57 (1.26, 1.96)	1.56 (1.27, 1.93)
*p*-value for trend		<0.001	<0.001
Per 250 mg increase		1.13 (1.06, 1.22)	

Notes: aOR: adjusted odds ratios; rOR: adjusted odds ratios with residual method; SFAs: saturated fatty acids; MUFAs: monounsaturated fatty acids; PUFAs: polyunsaturated fatty acids; n-6 PUFAs: including LA and AA; LA: linoleic acid; AA: arachidonic acid; n-3 PUFAs: including ALA, EPA, and DHA; ALA: α-linolenic acid; EPA: eicosapentaenoic acid; DHA: docosahexaenoic acid; ^a^ adjusted for study area, age (continuous), gender (male/female), education level (illiterate, primary school, middle school, high school or above), income 10 years ago (<1000, 1000 to <1500, 1500 to <2500, ≥2500), smoking (continuous, pack-years), alcohol consumption (continuous, g ethanol/day), family history of stomach cancer (yes/no), *H. pylori* infection (positive/negative), BMI (<18.5, 18.5 to <24, 24 to <28, ≥28), exercise 10 years ago (yes/no), dietary sodium intake (quartile levels of raw for aOR and energy-adjusted for rOR) and total energy intake (continuous, kcal/day); ^b^ The rescaling units for the continuous variables were chosen based on the interquartile range (IQR) of the controls and feasible intervention ranges.

**Table 4:** The interactions between dietary intakes of saturated, monounsaturated fatty acids, and total cholesterol and main risk factors on stomach cancer.

		Cases/Controls	aOR (95% CI) ^a^	rOR (95% CI) ^a^	RERI	ROR
Tobacco Smoking						
Non-smoker	Low SFAs	302/1611	1.00	1.00	RERI: −0.27 (−0.67, 0.13)	ROR: 0.76 (0.59, 0.99)
Non-smoker	High SFAs	432/1525	1.47 (1.18, 1.84)	1.20 (0.98, 1.47)	rRERI: 0.10 (−0.24, 0.43)	rROR: 1.01 (0.78, 1.31)
Smoker	Low SFAs	497/1655	1.65 (1.34, 2.04)	1.41 (1.16, 1.73)		
Smoker	High SFAs	669/1741	1.86 (1.49, 2.32)	1.71 (1.39, 2.10)		
Non-smoker	Low MUFAs	298/1597	1.00	1.00	RERI: −0.17 (−0.57, 0.23)	ROR: 0.80 (0.62, 1.04)
Non-smoker	High MUFAs	436/1539	1.50 (1.20, 1.87)	1.16 (0.95, 1.43)	rRERI: 0.17 (−0.16, 0.49)	rROR: 1.07 (0.82, 1.38)
Smoker	Low MUFAs	493/1669	1.61 (1.30, 1.99)	1.37 (1.12, 1.68)		
Smoker	High MUFAs	673/1727	1.94 (1.55, 2.42)	1.70 (1.38, 2.09)		
Non-smoker	Low Cholesterol	325/1600	1.00	1.00	RERI: −0.05 (−0.43, 0.34)	ROR: 0.87 (0.67, 1.12)
Non-smoker	High Cholesterol	409/1536	1.47 (1.20, 1.80)	1.50 (1.22, 1.83)	rRERI: −0.16 (−0.55, 0.24)	rROR: 0.81 (0.63, 1.05)
Smoker	Low Cholesterol	511/1666	1.53 (1.25, 1.87)	1.58 (1.30, 1.93)		
Smoker	High Cholesterol	655/1730	1.95 (1.58, 2.40)	1.92 (1.56, 2.36)		
Alcohol Drinking						
Non-drinker	Low SFAs	429/1943	1.00	1.00	RERI: −0.34 (−0.69, 0.01)	ROR: 0.75 (0.57, 0.98)
Non-drinker	High SFAs	605/1749	1.40 (1.14, 1.72)	1.16 (0.96, 1.40)	rRERI: 0.05 (−0.24, 0.34)	rROR:1.04 (0.80, 1.35)
Drinker	Low SFAs	370/1323	1.23 (0.99, 1.53)	1.03 (0.84, 1.26)		
Drinker	High SFAs	496/1517	1.29 (1.03, 1.61)	1.24 (1.00, 1.54)		
Non-drinker	Low MUFAs	429/1927	1.00	1.00	RERI: −0.26 (−0.60, 0.09)	ROR: 0.80 (0.61, 1.05)
Non-drinker	High MUFAs	605/1765	1.42 (1.16, 1.74)	1.16 (0.96, 1.40)	rRERI: 0.06 (−0.23, 0.35)	rROR: 1.05 (0.80, 1.37)
Drinker	Low MUFAs	362/1339	1.19 (0.95, 1.47)	1.03 (0.84, 1.26)		
Drinker	High MUFAs	504/1501	1.35 (1.08, 1.69)	1.25 (1.01, 1.55)		
Non-drinker	Low Cholesterol	463/1932	1.00	1.00	RERI: −0.30 (−0.66, 0.06)	ROR: 0.78 (0.60, 1.02)
Non-drinker	High Cholesterol	571/1760	1.53 (1.26, 1.84)	1.45 (1.21, 1.74)	rRERI: −0.18 (−0.53, 0.17)	rROR: 0.85 (0.65, 1.11)
Drinker	Low Cholesterol	373/1334	1.18 (0.96, 1.45)	1.14 (0.93, 1.38)		
Drinker	High Cholesterol	493/1506	1.41 (1.14, 1.75)	1.41 (1.14, 1.74)		
*H. pylori* Infection						
Negative	Low SFAs	139/861	1.00	1.00	RERI: −0.10 (−0.51, 0.31)	ROR:0.87 (0.64, 1.18)
Negative	High SFAs	193/752	1.40 (1.05, 1.86)	1.24 (0.94, 1.63)	rRERI: 0.02 (−0.35, 0.39)	rROR:0.97 (0.71, 1.31)
Positive	Low SFAs	436/1873	1.40 (1.11, 1.77)	1.32 (1.05, 1.67)		
Positive	High SFAs	626/1938	1.70 (1.33, 2.18)	1.59 (1.26, 2.00)		
Negative	Low MUFAs	138/839	1.00	1.00	RERI: 0.00 (−0.40, 0.39)	ROR:0.92 (0.68, 1.26)
Negative	High MUFAs	194/774	1.40 (1.05, 1.86)	1.17 (0.89, 1.53)	rRERI: 0.13 (−0.22, 0.47)	rROR:1.06 (0.78, 1.44)
Positive	Low MUFAs	428/1869	1.35 (1.07, 1.71)	1.26 (1.00, 1.58)		
Positive	High MUFAs	634/1942	1.75 (1.37, 2.24)	1.55 (1.23, 1.95)		
Negative	Low Cholesterol	134/782	1.00	1.00	RERI: −0.23 (−0.69, 0.24)	ROR: 0.78 (0.57, 1.06)
Negative	High Cholesterol	198/831	1.66 (1.26, 2.19)	1.68 (1.28, 2.22)	rRERI: −0.26 (−0.73, 0.22)	rROR: 0.76 (0.56, 1.04)
Positive	Low Cholesterol	491/1990	1.49 (1.18, 1.89)	1.50 (1.19, 1.90)		
Positive	High Cholesterol	571/1821	1.93 (1.52, 2.45)	1.93 (1.52, 2.44)		
Dietary Sodium Intake						
Low sodium	Low SFAs	500/2203	1.00	1.00	RERI: −0.33 (−0.73, 0.05)	ROR: 0.75 (0.57, 0.99)
Low sodium	High SFAs	371/1063	1.48 (1.20, 1.83)	1.37 (1.12, 1.68)	rRERI: −0.25 (−0.61, 0.12)	rROR: 0.80 (0.61, 1.05)
High sodium	Low SFAs	299/1063	1.31 (1.06, 1.62)	1.29 (1.06, 1.57)		
High sodium	High SFAs	730/2203	1.45 (1.20, 1.77)	1.41 (1.18, 1.69)		
Low sodium	Low MUFAs	495/2217	1.00	1.00	RERI: −0.44 (−0.86, −0.03)	ROR: 0.70 (0.53, 0.92)
Low sodium	High MUFAs	376/1049	1.61 (1.30, 1.98)	1.35 (1.10, 1.65)	rRERI: −0.19 (−0.55, 0.17)	rROR: 0.83 (0.63, 1.10)
High sodium	Low MUFAs	296/1049	1.35 (1.09, 1.67)	1.26 (1.03, 1.53)		
High sodium	High MUFAs	733/2217	1.51 (1.25, 1.83)	1.42 (1.18, 1.69)		
Low sodium	Low Cholesterol	495/2108	1.00	1.00	RERI: −0.07 (−0.43, 0.30)	ROR: 0.92 (0.70, 1.21)
Low sodium	High Cholesterol	376/1158	1.45 (1.18, 1.79)	1.43 (1.17, 1.76)	rRERI: −0.03 (−0.39, 0.33)	rROR: 0.94 (0.71, 1.23)
High sodium	Low Cholesterol	341/1158	1.15 (0.94, 1.40)	1.18 (0.97, 1.43)		
High sodium	High Cholesterol	688/2108	1.53 (1.27, 1.84)	1.59 (1.32, 1.91)		
Family history of stomach cancer						
No	Low SFAs	728/3120	1.00	1.00	RERI: 0.17 (−0.73, 1.07)	ROR: 0.99 (0.62, 1.59)
No	High SFAs	983/3050	1.26 (1.06, 1.49)	1.19 (1.02, 1.39)	rRERI: 0.43 (−0.45, 1.31)	rROR: 1.16 (0.73, 1.83)
Yes	Low SFAs	71/146	1.77 (1.21, 2.58)	1.64 (1.18, 2.28)		
Yes	High SFAs	118/216	2.20 (1.60, 3.03)	2.26 (1.62, 3.16)		
No	Low MUFAs	720/3111	1.00	1.00	RERI: 0.36 (−0.55, 1.28)	ROR: 1.07 (0.66, 1.71)
No	High MUFAs	991/3059	1.31 (1.11, 1.55)	1.20 (1.03, 1.40)	rRERI: 0.34 (−0.53, 1.22)	rROR: 1.10 (0.70, 1.75)
Yes	Low MUFAs	71/155	1.70 (1.16, 2.47)	1.67 (1.20, 2.33)		
Yes	High MUFAs	118/207	2.37 (1.72, 3.26)	2.22 (1.59, 3.09)		
No	Low Cholesterol	741/3066	1.00	1.00	RERI: 0.46 (−0.51, 1.42)	ROR: 1.09 (0.69, 1.73)
No	High Cholesterol	970/3104	1.36 (1.17, 1.59)	1.33 (1.14, 1.55)	rRERI: 0.99 (−0.15, 2.13)	rROR: 1.38 (0.86, 2.22)
Yes	Low Cholesterol	95/200	1.69 (1.22, 2.33)	1.58 (1.17, 2.12)		
Yes	High Cholesterol	94/162	2.51 (1.78, 3.53)	2.90 (1.99, 4.21)		

Notes: aOR: adjusted odds ratios; rOR: adjusted odds ratios with residual method; RERI: the relative excess risk due to interaction; ROR: the ratio of odds ratio; rRERI: RERI with residual method; rROR: ROR with residual method; SFAs: saturated fatty acids; MUFAs: monounsaturated fatty acids; ^a^ Adjusted for study area, age (continuous), gender (male/female), education level (illiterate, primary school, middle school, high school or above), income 10 years ago (<1000, 1000 to <1500, 1500 to<2500, ≥2500), smoking (continuous, pack-years), alcohol consumption (continuous, g ethanol/day), *H. pylori* infection (positive/negative), family history of stomach cancer (yes/no), BMI (<18.5, 18.5 to <24, 24 to <28, ≥28), exercise 10 years ago (yes/no), dietary sodium intake (quartile levels of raw and energy-adjusted values), total energy intake (continuous, kcal/day), except for the corresponding variables used for interaction.

**Table 5:** The associations between dietary saturated, monounsaturated fatty acids, total cholesterol (high vs. low) and stomach cancer, stratified by tobacco smoking, alcohol drinking, *H. pylori* infection, and dietary sodium intake.

Stratum	aOR (95% CI) *		*p* ^†^	rOR (95% CI) *		*p* ^†^
Tobacco smoking	Never-smokers	Ever-smokers		Never-smokers	Ever-smokers	
SFAs	1.43 (1.11, 1.83)	1.13 (0.92, 1.39)	0.04	1.21 (0.97, 1.50)	1.20 (1.00, 1.45)	0.95
MUFAs	1.43 (1.12, 1.82)	1.22 (1.00, 1.50)	0.10	1.16 (0.93, 1.44)	1.24 (1.03, 1.49)	0.63
Total cholesterol	1.36 (1.10, 1.70)	1.34 (1.11, 1.62)	0.28	1.46 (1.18, 1.81)	1.24 (1.03, 1.49)	0.11
Alcohol drinking	Non-drinkers	Drinkers		Non-drinkers	Drinkers	
SFAs	1.33 (1.07, 1.66)	1.13 (0.88, 1.45)	0.03	1.18 (0.97, 1.43)	1.17 (0.94, 1.47)	0.79
MUFAs	1.33 (1.07, 1.66)	1.24 (0.97, 1.59)	0.11	1.18 (0.97, 1.44)	1.18 (0.94, 1.47)	0.73
Total cholesterol	1.49 (1.22, 1.82)	1.24 (0.99, 1.55)	0.07	1.49 (1.23, 1.81)	1.20 (0.96, 1.50)	0.24
*H. pylori* infection	Negative	Positive		Negative	Positive	
SFAs	1.50 (1.06, 2.11)	1.17 (0.97, 1.42)	0.36	1.27 (0.94, 1.71)	1.19 (1.01, 1.41)	0.83
MUFAs	1.46 (1.04, 2.05)	1.26 (1.05, 1.52)	0.62	1.15 (0.85, 1.56)	1.23 (1.04, 1.46)	0.72
Total cholesterol	1.86 (1.37, 2.54)	1.26 (1.07, 1.50)	0.11	1.85 (1.36, 2.50)	1.25 (1.06, 1.48)	0.09
Dietary sodium intake	Low	High		Low	High	
SFAs	1.40 (1.11, 1.76)	1.16 (0.92, 1.46)	0.04	1.40 (1.14, 1.72)	1.05 (0.85, 1.29)	0.12
MUFAs	1.54 (1.23, 1.94)	1.16 (0.92, 1.45)	0.01	1.38 (1.12, 1.70)	1.08 (0.88, 1.33)	0.21
Total cholesterol	1.39 (1.12, 1.73)	1.35 (1.10, 1.66)	0.55	1.44 (1.16, 1.77)	1.32 (1.08, 1.61)	0.64
Family history of stomach cancer	No	Yes		No	Yes	
SFAs	1.27 (1.06, 1.51)	1.07 (0.58, 1.97)	0.96	1.20 (1.03, 1.40)	1.39 (0.82, 2.34)	0.54
MUFAs	1.31 (1.11, 1.56)	1.30 (0.71, 2.36)	0.79	1.21 (1.04, 1.41)	1.35 (0.80, 2.26)	0.68
Total cholesterol	1.37 (1.17, 1.60)	1.51 (0.89, 2.54)	0.71	1.34 (1.15, 1.56)	1.74 (1.04, 2.91)	0.18

Notes: aOR: adjusted odds ratios; rOR: adjusted odds ratios with residual method; SFAs: saturated fatty acids; MUFAs: monounsaturated fatty acids. * Adjusted for study area, age (continuous), gender (male/female), education level (illiterate, primary school, middle school, high school or above), income 10 years ago (<1000, 1000 to <1500, 1500 to <2500, ≥2500), smoking (continuous, pack-years), alcohol consumption (continuous, g ethanol/day), *H. pylori* infection (positive/negative), family history of stomach cancer (yes/no), BMI (<18.5, 18.5 to <24, 24 to <28, ≥28), exercise 10 years ago (yes/no), dietary sodium intake (quartile levels of raw and energy-adjusted values), total energy intake (continuous, kcal/day), except for the corresponding variable used for stratification. ^†^ Test for heterogeneity and *p* < 0.1 was considered statistically significant.

## Supplementary Materials

The following are available online at https://www.mdpi.com/2072-6643/11/8/1730/s1, Table S1: The crude odds ratios of main risk factors on stomach cancer (*n* = 10,235); Table S2: Adjusted odds ratios and 95% CIs for associations between dietary fatty acids, total cholesterol and stomach cancer stratified by proxy interview (non-proxy = 7383, proxy = 1049); Table S3: The associations between dietary fatty acids, total cholesterol, and stomach cancer, excluding individuals with reported total energy intake in the upper and lower 2.5% (cases = 1907, controls = 6598); Table S4: Adjusted odds ratios of stomach cancer for daily intakes of fatty acids and total cholesterol using multiple imputations and semi-Bayes shrinkage methods; Figure S1: The study flowchart showing sample sizes.
