# Supplementary material for: Dietary Intake of Fatty Acids, Total Cholesterol, and Stomach Cancer in a Chinese Population
Source: Nutrients. 2019 Jul 26;11(8):1730. doi: 10.3390/nu11081730 (PMC6723637; doi:10.3390/nu11081730)

Supplemental Table 1. The crude odds ratios of main risk factors on stomach cancer (n=10,235).

**Variables**	**Cases(n=2216)**	**Controls (n=8019)**	**cOR (95%CI)**
Gender			
Male	1632 (73.7)	5767 (71.9)	1.09 (0.98, 1.21)
Female	584 (26.4)	2252 (28.1)	1.00
Age			
<50	237 (10.7)	884 (11.0)	1.00
50-59	473 (21.3)	1794 (22.4)	0.98 (0.83, 1.17)
60-69	745 (33.6)	2565 (32.0)	1.08 (0.92, 1.28)
>=70	761 (34.3)	2776 (34.6)	1.02 (0.87, 1.21)
Education			
Illiterate	1096 (49.6)	3831 (47.8)	1.00
Primary school	773 (34.9)	2515 (31.4)	1.07 (0.97, 1.19)
Middle school	279 (12.6)	1320 (16.5)	**0.74 (0.64, 0.85)**
High school or above	62 (2.8)	335 (4.2)	**0.65 (0.49, 0.86)**
Income 10 years ago (Yuan/year)			
<1000	525 (23.7)	1710 (21.3)	1.00
1000-1499	437 (19.7)	1530 (19.1)	0.93 (0.81, 1.07)
1500-2499	563 (25.4)	2074 (25.9)	0.88 (0.77, 1.01)
>=2500	630 (28.4)	2572 (32.1)	**0.80 (0.70, 0.91)**
Body mass index (kg/m2)			
<18.5	327 (14.8)	454 (5.7)	**2.35 (2.02, 2.75)**
18.5-<24	1480 (66.8)	4839 (60.3)	1.00
24-<28	315 (14.2)	2230 (27.8)	**0.46 (0.40, 0.53)**
>=28	70 (3.2)	453 (5.7)	**0.51 (0.39, 0.65)**
Exercise ten years ago			
No	1364 (61.6)	4723 (58.9)	1.00
Yes	852 (38.5)	3296 (41.1)	**0.90 (0.81, 0.99)**
Pack-years of tobacco smoking			
0	923 (41.7)	4292 (53.5)	1.00
1 to 19	255 (11.5)	811 (10.1)	**1.46 (1.25, 1.71)**
20-39	339 (15.3)	1133 (14.1)	**1.39 (1.21, 1.60)**
>=40	510 (23.0)	1241 (15.5)	**1.91 (1.69, 2.17)**
Grams ethanol/week in the 1990's*			
Never	1288 (58.1)	5054 (63.0)	1.00
Low-risk	151 (6.8)	533 (6.7)	1.11 (0.92, 1.35)
High-risk	745 (33.6)	2279 (28.4)	**1.28 (1.16, 1.42)**
Family history of stomach cancer			
No	2022 (91.2)	7644 (95.3)	1.00
Yes	194 (8.8)	375 (4.7)	**1.96 (1.63, 2.34)**
H. Pylori infection			
No	377 (17.1)	1918 (23.9)	1.00
Yes	1247 (56.3)	4748 (59.2)	**1.34 (1.18, 1.52)**

Notes: cOR: crude odds ratios;
*: Low-risk drinking: men (≤ 25 g ethanol /day) and women (≤ 15 g ethanol /day); High-risk drinking: men (>25 g ethanol /day) and women (>15 g ethanol /day based on the 2016 Chinese Dietary Guide.

Supplemental Table 2. Adjusted odds ratios and 95% CIs for associations between dietary fatty acids, total cholesterol and stomach cancer in Jiangsu study stratified by proxy interview (Non-proxy =7,383, Proxy=1,049).

	Non-proxy (n=7,383)			Proxy (n=1,049)		
Variables	Cases/controls	aOR (95%CI)[a]	rOR (95%CI)[a]	Cases/controls	aOR (95%CI)[a]	rOR (95%CI)[a]
Total fatty acid (g/day)						
Q1 (<14.85)	230/1472	1.00	1.00	126/161	1.00	1.00
Q2 (14.85-<24.75)	286/1527	**1.32 (1.03, 1.71)**	1.10 (0.87, 1.38)	145/106	1.12 (0.68, 1.84)	1.40 (0.80, 2.44)
Q3 (24.75-<40.17)	408/1527	**1.59 (1.22, 2.07)**	**1.29 (1.02, 1.63)**	173/106	1.38 (0.82, 2.34)	1.32 (0.76, 2.28)
Q4 (40.17+)	403/1530	**1.46 (1.07, 1.99)**	1.06 (0.83, 1.35)	129/103	1.34 (0.68, 2.65)	1.21 (0.67, 2.16)
P value for trend		**0.01**	0.44		0.27	0.7
Per 25g increase		1.06 (0.95, 1.18)			1.03 (0.81, 1.32)	
SFAs (g/day)						
Q1 (<4.35)	232/1480	1.00	1.00	129/153	1.00	1.00
Q2 (4.35-<7.14)	294/1518	**1.30 (1.01, 1.67)**	1.08 (0.86, 1.37)	144/115	0.98 (0.60, 1.60)	1.33 (0.77, 2.28)
Q3 (7.14-<11.56)	402/1535	**1.45 (1.12, 1.89)**	**1.32 (1.04, 1.67)**	164/98	1.38 (0.81, 2.33)	1.09 (0.63, 1.89)
Q4 (11.56+)	399/1523	**1.37 (1.00, 1.86)**	1.15 (0.90, 1.47)	136/110	1.20 (0.63, 2.30)	1.21 (0.69, 2.15)
P value for trend		**0.046**	0.13		0.33	0.76
Per 7g increase		1.07 (0.96, 1.19)			1.05 (0.84, 1.32)	
MUFAs (g/day)						
Q1 (<5.57)	230/1488	1.00	1.00	126/145	1.00	1.00
Q2 (5.57-<9.85)	285/1509	1.20 (0.93, 1.54)	1.08 (0.86, 1.36)	150/124	0.90 (0.56, 1.45)	1.10 (0.63, 1.92)
Q3 (9.85-<16.65)	406/1539	**1.55 (1.20, 2.01)**	**1.35 (1.07, 1.71)**	166/94	1.16 (0.69, 1.95)	1.32 (0.76, 2.29)
Q4 (16.65+)	406/1520	**1.48 (1.10, 1.99)**	1.09 (0.85, 1.40)	131/113	0.91 (0.48, 1.72)	1.05 (0.59, 1.87)
P value for trend		**0.003**	0.25		0.93	0.75
Per 10g increase		1.03 (0.94, 1.13)			1.00 (0.82, 1.22)	
PUFAs (g/day)						
Q1 (<4.28)	239/1463	1.00	1.00	126/170	1.00	1.00
Q2 (4.28-<6.93)	301/1524	1.11 (0.86, 1.44)	1.11 (0.88, 1.39)	160/109	1.55 (0.95, 2.54)	1.12 (0.65, 1.94)
Q3 (6.93-<10.80)	394/1532	**1.34 (1.03, 1.75)**	0.99 (0.78, 1.26)	166/101	1.63 (0.92, 2.88)	1.51 (0.86, 2.64)
Q4 (10.80+)	393/1537	1.12 (0.81, 1.54)	1.11 (0.88, 1.40)	121/96	1.52 (0.73, 3.17)	1.10 (0.61, 1.98)
P value for trend		0.34	0.59		0.21	0.53
Per 6g increase		1.07 (0.96, 1.20)			1.08 (0.85, 1.38)	

n-6 PUFAs (g/day)						
Q1 (<3.76)	244/1458	1.00	1.00	128/175	1.00	1.00
Q2 (3.76-<5.97)	304/1529	1.15 (0.89, 1.48)	1.04 (0.83, 1.30)	166/104	**1.67 (1.01, 2.76)**	1.25 (0.72, 2.15)
Q3 (5.97-<9.14)	383/1530	1.25 (0.96, 1.64)	0.87 (0.68, 1.11)	155/103	1.45 (0.81, 2.57)	1.53 (0.88, 2.68)
Q4 (9.14+)	396/1539	1.13 (0.82, 1.56)	1.12 (0.89, 1.40)	124/94	1.61 (0.78, 3.36)	1.25 (0.69, 2.26)
P value for trend		0.42	0.56		0.26	0.37
Per 5g increase		1.07 (0.96, 1.20)			1.11 (0.87, 1.42)	
LA (g/day)						
Q1 (<3.49)	251/1463	1.00	1.00	129/170	1.00	1.00
Q2 (3.49-<5.50)	310/1521	1.12 (0.87, 1.44)	1.03 (0.82, 1.29)	164/112	1.46 (0.89, 2.38)	1.16 (0.67, 1.99)
Q3 (5.50-<8.35)	369/1532	1.09 (0.83, 1.43)	0.88 (0.70, 1.12)	153/101	1.56 (0.87, 2.80)	1.29 (0.73, 2.28)
Q4 (8.35+)	397/1540	1.08 (0.79, 1.49)	1.13 (0.90, 1.42)	127/93	1.58 (0.76, 3.30)	1.23 (0.67, 2.23)
P value for trend		0.72	0.47		0.2	0.47
Per 5g increase		1.10 (0.97, 1.24)			1.14 (0.87, 1.49)	
AA (g/day)						
Q1 (<0.18)	253/1496	1.00	1.00	141/137	1.00	1.00
Q2 (0.18-<0.38)	292/1502	1.13 (0.88, 1.44)	1.03 (0.81, 1.30)	146/131	0.87 (0.55, 1.39)	1.05 (0.61, 1.82)
Q3 (0.38-<0.78)	393/1536	**1.34 (1.04, 1.71)**	**1.27 (1.00, 1.62)**	160/97	0.91 (0.55, 1.50)	0.86 (0.50, 1.50)
Q4 (0.78+)	389/1522	1.16 (0.88, 1.53)	0.95 (0.75, 1.21)	126/111	1.00 (0.57, 1.75)	1.00 (0.57, 1.75)
P value for trend		0.19	0.94		0.98	0.78
Per 1g increase		0.95 (0.82, 1.10)			0.96 (0.72, 1.27)	
n-3 PUFAs (g/day)						
Q1 (<0.54)	241/1473	1.00	1.00	137/160	1.00	1.00
Q2 (0.54-<0.96)	276/1520	1.09 (0.85, 1.41)	1.11 (0.88, 1.40)	141/113	0.97 (0.60, 1.58)	1.14 (0.67, 1.93)
Q3 (0.96-<1.70)	410/1533	**1.43 (1.10, 1.85)**	1.21 (0.95, 1.53)	185/100	1.37 (0.81, 2.32)	1.31 (0.77, 2.24)
Q4 (1.70+)	400/1530	1.20 (0.89, 1.62)	1.05 (0.83, 1.33)	110/103	0.86 (0.44, 1.69)	1.08 (0.61, 1.93)
P value for trend		0.1	0.61		0.83	0.67
Per 1g increase		1.02 (0.93, 1.12)			0.99 (0.81, 1.20)	
ALA (g/day)						
Q1 (<0.52)	247/1474	1.00	1.00	133/159	1.00	1.00
Q2 (0.52-<0.93)	270/1523	1.04 (0.81, 1.35)	1.08 (0.86, 1.37)	150/110	1.03 (0.63, 1.68)	1.18 (0.69, 2.00)
Q3 (0.93-<1.64)	411/1533	**1.40 (1.09, 1.81)**	1.22 (0.97, 1.55)	178/100	1.35 (0.80, 2.27)	1.36 (0.79, 2.33)

Q4 (1.64+)	399/1526	1.17 (0.87, 1.57)	1.01 (0.80, 1.28)	112/107	0.83 (0.43, 1.61)	1.13 (0.64, 2.00)
P value for trend		0.12	0.76		0.95	0.58
Per 1g increase		1.02 (0.92, 1.12)			0.99 (0.81, 1.21)	
EPA (mg/day)						
Q1 (<4.30)	253/1449	1.00	1.00	145/184	1.00	1.00
Q2 (4.30-<10.47)	332/1523	1.10 (0.86, 1.41)	0.93 (0.73, 1.17)	171/110	**1.98 (1.26, 3.12)**	1.01 (0.61, 1.68)
Q3 (10.47-<21.77)	327/1534	1.09 (0.84, 1.42)	0.99 (0.77, 1.27)	123/99	1.31 (0.78, 2.21)	0.87 (0.51, 1.50)
Q4 (21.77+)	415/1550	1.18 (0.89, 1.56)	1.13 (0.89, 1.42)	134/83	1.51 (0.85, 2.66)	1.22 (0.71, 2.11)
P value for trend		0.31	0.23		0.24	0.59
Per 20mg increase		1.01 (0.98, 1.05)			0.98 (0.85, 1.12)	
DHA (mg/day)						
Q1 (<2.52)	248/1435	1.00	1.00	175/198	1.00	1.00
Q2 (2.52-<9.97)	307/1528	1.04 (0.80, 1.34)	0.87 (0.68, 1.11)	141/105	**2.01 (1.25, 3.23)**	1.05 (0.63, 1.76)
Q3 (9.97-<24.49)	318/1547	0.97 (0.73, 1.28)	0.87 (0.67, 1.12)	125/86	**1.86 (1.08, 3.20)**	0.89 (0.51, 1.56)
Q4 (24.49+)	454/1546	1.28 (0.95, 1.71)	1.12 (0.89, 1.42)	132/87	**1.88 (1.05, 3.35)**	1.45 (0.83, 2.55)
P value for trend		0.08	0.22		**0.03**	0.28
Per 20mg increase		**1.06 (1.02, 1.10)**			1.01 (0.88, 1.15)	
n-3/n-6 PUFAs						
Q1 (<0.13)	262/1498	1.00	1.00	144/135	1.00	1.00
Q2 (0.13-<0.16)	309/1528	0.92 (0.73, 1.18)	0.88 (0.70, 1.11)	140/105	1.24 (0.76, 2.03)	0.76 (0.45, 1.30)
Q3 (0.16-<0.20)	380/1535	**1.30 (1.03, 1.63)**	1.12 (0.89, 1.41)	155/98	1.09 (0.67, 1.78)	0.81 (0.48, 1.38)
Q4 (0.20+)	376/1495	1.14 (0.89, 1.46)	1.11 (0.88, 1.38)	134/138	0.73 (0.45, 1.19)	0.64 (0.38, 1.06)
P value for trend		0.06	0.15		0.19	0.12
Per 0.1 increase		1.11 (0.96, 1.30)			0.95 (0.70, 1.29)	
Total fat (g/day)						
Q1 (<18.84)	239/1487	1.00	1.00	116/146	1.00	1.00
Q2 (18.84-<31.07)	314/1510	**1.29 (1.01, 1.65)**	1.14 (0.91, 1.43)	149/123	1.03 (0.64, 1.68)	0.95 (0.54, 1.67)
Q3 (31.07-<48.98)	389/1533	**1.41 (1.09, 1.82)**	1.14 (0.89, 1.44)	168/100	1.15 (0.66, 1.99)	1.22 (0.69, 2.15)
Q4 (48.98+)	385/1526	1.26 (0.93, 1.71)	1.02 (0.80, 1.31)	140/107	1.15 (0.58, 2.27)	0.97 (0.52, 1.79)
P value for trend		0.12	0.89		0.62	0.82
Per 30g increase		1.03 (0.92, 1.16)			0.95 (0.74, 1.23)	
Total cholesterol						

(mg/day)						
Q1 (<107.24)	300/1506	1.00	1.00	107/127	1.00	1.00
Q2 (107.24-<207.21)	318/1503	1.06 (0.84, 1.33)	1.05 (0.85, 1.31)	111/130	0.87 (0.53, 1.42)	**2.30 (1.32, 3.99)**
Q3 (207.21-<352.09)	344/1534	1.20 (0.95, 1.51)	**1.28 (1.01, 1.61)**	154/99	**1.76 (1.05, 2.93)**	**2.30 (1.34, 3.96)**
Q4 (352.09+)	365/1513	**1.52 (1.17, 1.96)**	**1.46 (1.15, 1.86)**	201/120	**1.89 (1.08, 3.30)**	**2.37 (1.36, 4.16)**
P value for trend		**0.001**	**0.001**		**0.005**	**0.007**
Per 250mg increase		**1.13 (1.04, 1.22)**			**1.26 (1.02, 1.55)**	

Notes: aOR: adjusted odds ratios; rOR: adjusted odds ratios with residual methods; SFAs: Saturated fatty acids; MUFAs: Monounsaturated fatty acids; PUFAs: Polyunsaturated fatty acids; n-6 PUFAs: including LA and AA; LA: Linoleic acid; AA: Arachidonic acid; n-3 PUFAs: including ALA, EPA and DHA; ALA: α-linolenic acid; EPA: Eicosapentaenoic acid; DHA: Docosahexaenoic acid.

[a]: Adjusted for study area, age, gender, education level, income, smoking, drinking, family history of stomach cancer, H. pylori infection, body mass index, exercise ten years ago, dietary sodium intake and total energy intake.

Supplemental Table 3. The associations between dietary fatty acids, total cholesterol and stomach cancer excluding individuals with reported total energy intake in the upper and lower 2.5% (cases=1,907, controls=6,598).

**Variables**	**Cases/Controls**	**aOR (95%CI)[a]**	**rOR (95%CI)[a]**
Total fatty acid (g/day)			
Q1	359/1649	1.00	1.00
Q2	428/1650	1.16 (0.93, 1.43)	1.19 (0.97, 1.45)
Q3	589/1650	**1.52 (1.22, 1.90)**	**1.33 (1.08, 1.63)**
Q4	531/1649	**1.34 (1.03, 1.75)**	1.19 (0.96, 1.47)
P for trend		**0.006**	0.075
SFAs (g/day)			
Q1	358/1649	1.00	1.00
Q2	439/1650	**1.24 (1.00, 1.54)**	**1.25 (1.02, 1.53)**
Q3	572/1650	**1.51 (1.20, 1.88)**	**1.35 (1.10, 1.66)**
Q4	538/1649	**1.46 (1.12, 1.89)**	**1.27 (1.03, 1.57)**
P for trend		**0.002**	**0.024**
MUFAs (g/day)			
Q1	361/1650	1.00	1.00
Q2	435/1649	1.23 (0.99, 1.52)	1.21 (0.99, 1.48)
Q3	574/1649	**1.49 (1.20, 1.85)**	**1.43 (1.16, 1.75)**
Q4	537/1650	**1.44 (1.11, 1.85)**	1.21 (0.97, 1.49)
P for trend		**0.002**	**0.044**
PUFAs (g/day)			
Q1	375/1650	1.00	1.00
Q2	450/1649	1.12 (0.90, 1.39)	1.18 (0.96, 1.44)
Q3	557/1649	**1.40 (1.11, 1.76)**	1.21 (0.99, 1.49)
Q4	525/1650	1.17 (0.89, 1.53)	1.18 (0.96, 1.45)
P for trend		0.13	0.14
n-6 PUFAs (g/day)			
Q1	377/1650	1.00	1.00
Q2	473/1649	1.20 (0.97, 1.49)	1.18 (0.97, 1.44)
Q3	535/1650	**1.31 (1.04, 1.66)**	1.10 (0.90, 1.36)
Q4	522/1649	1.13 (0.86, 1.49)	1.16 (0.95, 1.42)
P for trend		0.35	0.27
LA (g/day)			
Q1	380/1650	1.00	1.00
Q2	478/1649	1.21 (0.98, 1.50)	1.17 (0.96, 1.43)
Q3	522/1649	1.25 (0.99, 1.58)	1.08 (0.88, 1.33)
Q4	527/1650	1.13 (0.86, 1.49)	1.17 (0.96, 1.43)
P for trend		0.45	0.24
AA (g/day)			
Q1	394/1649	1.00	1.00
Q2	441/1650	1.12 (0.91, 1.37)	1.15 (0.94, 1.40)
Q3	551/1649	**1.26 (1.02, 1.55)**	**1.27 (1.03, 1.56)**
Q4	521/1650	1.22 (0.97, 1.54)	1.11 (0.90, 1.36)
P for trend		0.06	0.27
n-3 PUFAs (g/day)			
Q1	375/1650	1.00	1.00
Q2	423/1649	1.15 (0.93, 1.43)	1.13 (0.93, 1.38)

Q3	593/1650	**1.47 (1.18, 1.83)**	**1.27 (1.04, 1.56)**
Q4	516/1649	1.25 (0.96, 1.61)	1.09 (0.89, 1.34)
P for trend		**0.03**	0.3
ALA (g/day)			
Q1	374/1649	1.00	1.00
Q2	429/1650	1.15 (0.93, 1.42)	1.16 (0.95, 1.42)
Q3	586/1649	**1.47 (1.18, 1.83)**	**1.29 (1.05, 1.58)**
Q4	518/1650	1.24 (0.96, 1.60)	1.13 (0.92, 1.38)
P for trend		**0.04**	0.21
EPA (mg/day)			
Q1	401/1650	1.00	1.00
Q2	503/1649	1.21 (0.99, 1.48)	0.94 (0.77, 1.15)
Q3	453/1650	1.09 (0.87, 1.35)	0.97 (0.79, 1.20)
Q4	550/1649	1.19 (0.94, 1.51)	1.08 (0.88, 1.32)
P for trend		0.35	0.42
DHA (mg/day)			
Q1	431/1646	1.00	1.00
Q2	447/1653	1.11 (0.90, 1.37)	**0.81 (0.66, 0.99)**
Q3	446/1649	1.03 (0.82, 1.30)	**0.80 (0.64, 0.99)**
Q4	583/1650	**1.33 (1.04, 1.69)**	1.10 (0.90, 1.35)
P for trend		**0.04**	0.23
n-3/n-6 PUFAs			
Q1	413/1649	1.00	1.00
Q2	450/1650	1.00 (0.81, 1.22)	0.94 (0.77, 1.14)
Q3	535/1650	**1.28 (1.05, 1.55)**	1.21 (0.99, 1.48)
Q4	509/1649	1.14 (0.93, 1.39)	1.10 (0.90, 1.33)
P for trend		0.05	0.1
Total fat (g/day)			
Q1	359/1649	1.00	1.00
Q2	462/1650	**1.31 (1.06, 1.61)**	**1.23 (1.01, 1.50)**
Q3	560/1649	**1.43 (1.14, 1.78)**	**1.27 (1.03, 1.56)**
Q4	526/1650	**1.32 (1.01, 1.71)**	1.12 (0.90, 1.40)
P for trend		**0.03**	0.32
Total cholesterol (mg/day)			
Q1	408/1649	1.00	1.00
Q2	436/1650	1.05 (0.86, 1.28)	1.16 (0.95, 1.40)
Q3	500/1650	**1.32 (1.08, 1.61)**	**1.39 (1.14, 1.70)**
Q4	563/1649	**1.52 (1.22, 1.90)**	**1.51 (1.23, 1.87)**
P for trend		**<0.001**	**<0.001**

Notes: aOR: adjusted odds ratios; rOR: adjusted odds ratios with residual methods; SFAs: Saturated fatty acids; MUFAs: Monounsaturated fatty acids; PUFAs: Polyunsaturated fatty acids; n-6 PUFAs: including LA and AA; LA: Linoleic acid; AA: Arachidonic acid; n-3 PUFAs: including ALA, EPA and DHA; ALA: α-linolenic acid; EPA: Eicosapentaenoic acid; DHA: Docosahexaenoic acid.

[a]: Adjusted for study area, age (continuous), gender (male/female), education level (illiterate, primary school, middle school, high school or above), income 10 years ago (<1000, 1000-1499, 1500-2499, ≥2500), smoking (continuous, pack-years), drinking (continuous, g ethanol/day), family history of stomach cancer (yes/no), H. *pylori* infection (positive/negative), BMI (<18.5, 18.5-24, 24-28, ≥28), exercise 10 years ago (yes/no), dietary sodium intake (quartile) and total energy intake (continuous, Kcal/day).

Supplemental Table 4. Adjusted odds ratios of stomach cancer for daily intakes of fatty acids and total cholesterol using multiple imputations and semi-Bayes shrinkage methods.

**Variables**	**miOR (95%CI)[a]**	**sbOR(95%CI)[a]**
Total fatty acid (g/day)		
Q1 (<14.85)	1.00	1.00
Q2 (14.85-<24.75)	0.95 (0.86, 1.05)	**1.24 (1.01, 1.52)**
Q3 (24.75-<40.17)	**1.18 (1.08, 1.30)**	**1.51 (1.22, 1.87)**
Q4 (40.17+)	1.12 (1.00, 1.27)	**1.37 (1.06, 1.76)**
P for trend	**<0.001**	
SFAs (g/day)		
Q1 (<4.35)	1.00	1.00
Q2 (4.35-<7.14)	0.95 (0.86, 1.04)	1.23 (1.00, 1.51)
Q3 (7.14-<11.56)	**1.16 (1.06, 1.26)**	**1.43 (1.16, 1.76)**
Q4 (11.56+)	1.11 (0.99, 1.24)	**1.36 (1.06, 1.74)**
P for trend	**<0.001**	
MUFAs (g/day)		
Q1 (<5.57)	1.00	1.00
Q2 (5.57-<9.85)	0.95 (0.86, 1.04)	1.16 (0.95, 1.43)
Q3 (9.85-<16.65)	**1.20 (1.10, 1.31)**	**1.45 (1.18, 1.78)**
Q4 (16.65+)	1.10 (0.99, 1.23)	**1.35 (1.06, 1.72)**
P for trend	**<0.001**	
PUFAs (g/day)		
Q1 (<4.28)	1.00	1.00
Q2 (4.28-<6.93)	1.00 (0.90, 1.10)	1.13 (0.92, 1.39)
Q3 (6.93-<10.80)	**1.14 (1.04, 1.26)**	**1.40 (1.13, 1.74)**
Q4 (10.80+)	1.03 (0.92, 1.16)	1.08 (0.83, 1.41)
P for trend	**0.03**	
Linoleic acid (g/day)		
Q1 (<3.49)	1.00	1.00
Q2 (3.49-<5.50)	1.02 (0.93, 1.12)	1.20 (0.98, 1.47)
Q3 (5.50-<8.35)	1.06 (0.97, 1.17)	1.22 (0.98, 1.52)
Q4 (8.35+)	1.03 (0.91, 1.16)	1.13 (0.87, 1.46)
P for trend	0.22	
AA (g/day)		
Q1 (<0.18)	1.00	1.00
Q2 (0.18-<0.38)	0.97 (0.87, 1.07)	1.09 (0.89, 1.33)
Q3 (0.38-<0.78)	**1.14 (1.04, 1.25)**	**1.22 (1.00, 1.49)**

Q4 (0.78+)	1.01 (0.91, 1.13)	1.13 (0.90, 1.42)
P for trend	0.08	
n-6 PUFAs (g/day)		
Q1 (<3.76)	1.00	1.00
Q2 (3.76-<5.97)	1.01 (0.92, 1.11)	1.17 (0.95, 1.43)
Q3 (5.97-<9.14)	1.10 (1.00, 1.20)	**1.31 (1.05, 1.63)**
Q4 (9.14+)	1.02 (0.90, 1.16)	1.12 (0.86, 1.45)
P for trend	0.15	
ALA (g/day)		
Q1 (<0.52)	1.00	1.00
Q2 (0.52-<0.93)	0.93 (0.85, 1.03)	1.05 (0.86, 1.29)
Q3 (0.93-<1.64)	**1.23 (1.12, 1.34)**	**1.40 (1.13, 1.72)**
Q4 (1.64+)	1.00 (0.89, 1.11)	1.10 (0.86, 1.40)
P for trend	**0.02**	
EPA (mg/day)		
Q1 (<4.30)	1.00	1.00
Q2 (4.30-<10.47)	1.04 (0.95, 1.14)	1.20 (0.99, 1.46)
Q3 (10.47-<21.77)	0.98 (0.89, 1.07)	1.07 (0.87, 1.32)
Q4 (21.77+)	**1.12 (1.01, 1.24)**	1.15 (0.92, 1.44)
P for trend	**0.02**	
DHA (mg/day)		
Q1 (<2.52)	1.00	1.00
Q2 (2.52-<9.97)	0.92 (0.84, 1.01)	1.10 (0.90, 1.34)
Q3 (9.97-<24.49)	0.99 (0.90, 1.08)	0.99 (0.80, 1.24)
Q4 (24.49+)	**1.23 (1.11, 1.37)**	**1.30 (1.03, 1.65)**
P for trend	**<0.001**	
n-3 PUFAs (g/day)		
Q1 (<0.54)	1.00	1.00
Q2 (0.54-<0.96)	0.92 (0.84, 1.02)	1.04 (0.85, 1.28)
Q3 (0.96-<1.70)	**1.24 (1.13, 1.35)**	**1.39 (1.13, 1.72)**
Q4 (1.70+)	1.00 (0.89, 1.13)	1.10 (0.86, 1.40)
P for trend	**0.01**	
n-3/n-6 PUFAs		
Q1 (<0.13)	1.00	1.00
Q2 (0.13-<0.16)	0.97 (0.89, 1.07)	0.97 (0.80, 1.18)
Q3 (0.16-<0.20)	**1.11 (1.01, 1.21)**	**1.24 (1.02, 1.49)**

Q4 (0.20+)	1.03 (0.94, 1.14)	1.08 (0.88, 1.31)
P for trend	0.054	
Total fat (g/day)		
Q1 (<18.84)	1.00	1.00
Q2 (18.84-<31.07)	1.01 (0.92, 1.11)	**1.28 (1.05, 1.56)**
Q3 (31.07-<48.98)	**1.14 (1.04, 1.25)**	**1.38 (1.12, 1.70)**
Q4 (48.98+)	1.05 (0.94, 1.18)	1.25 (0.98, 1.61)
P for trend	**0.007**	
Total cholesterol (mg/day)		
Q1 (<107.24)	1.00	1.00
Q2 (107.24-<207.21)	**0.89 (0.81, 0.98)**	1.06 (0.87, 1.28)
Q3 (207.21-<352.09)	1.04 (0.94, 1.14)	**1.29 (1.06, 1.57)**
Q4 (352.09+)	**1.20 (1.09, 1.33)**	**1.59 (1.29, 1.97)**
P for trend	**<0.001**	

Notes miOR: adjusted odds ratios using multiple imputations; sbOR: adjusted odds ratios with semi-Bayes shrinkage method; SFAs: Saturated fatty acids; MUFAs: Monounsaturated fatty acids; PUFAs: Polyunsaturated fatty acids; n-6 PUFAs: including LA and AA; LA: Linoleic acid; AA: Arachidonic acid; n-3 PUFAs: including ALA, EPA and DHA; ALA: α-linolenic acid; EPA: Eicosapentaenoic acid; DHA: Docosahexaenoic acid.

a: Adjusted for study area, age (continuous), gender (male/female), education level (illiterate, primary school, middle school, high school or above), income 10 years ago (<1000, 1000-1499, 1500-2499, ≥2500), smoking (continuous, pack-years), drinking (continuous, g ethanol/day), family history of stomach cancer (yes/no), H. *pylori* infection (positive/negative), BMI (<18.5, 18.5-24, 24-28, ≥28), exercise 10 years ago (yes/no), dietary sodium intake (quartile) and total energy intake (continuous, Kcal/day).

Supplemental Figure 1. The study flowchart showing sample size.

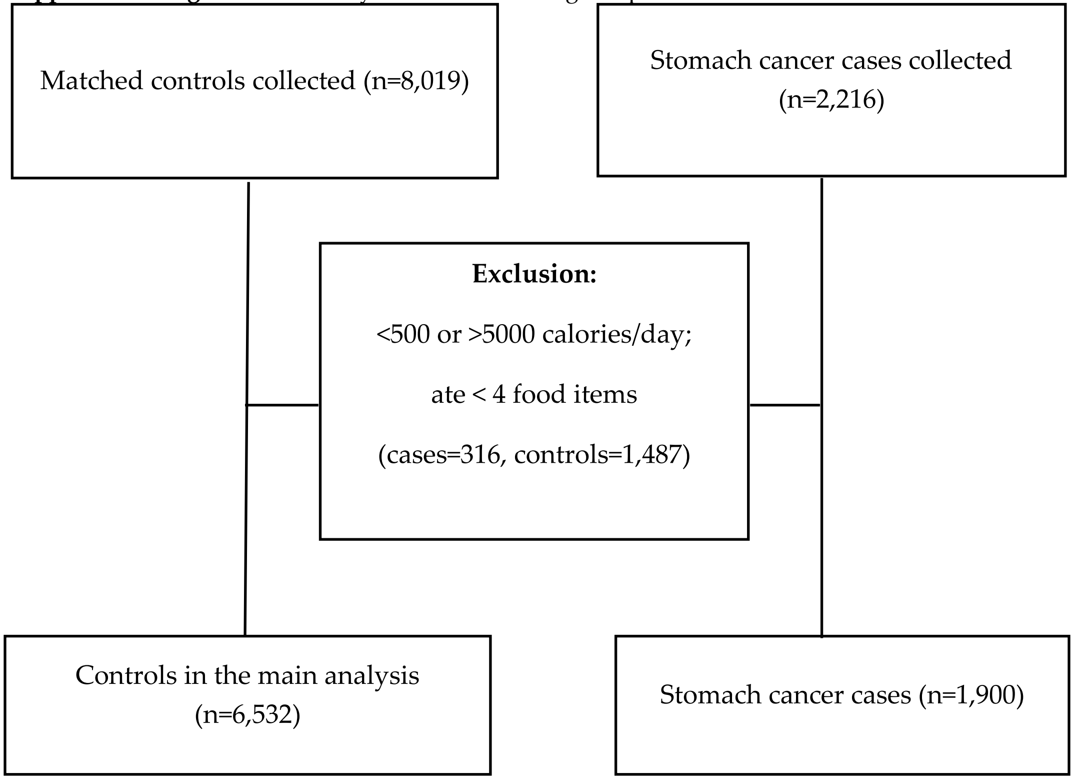

Supplement: Supplementary file 1 [file nutrients-11-01730-s001.pdf]
